# 

**Published:** 1867-09

**Authors:** 


### *Meeting of the Alumni of Rush Medical College.*

The enthusiastic response to the suggestion of a reunion of the Alumni of Rush Medical College, on the occasion of the opening of the new college building, has warranted those having the matter in charge to announce formally the time and place of meeting.

The Alumni will meet at the lower lecture-room of the new college, corner of Dearborn and Indiana Streets, Wednesday, October 2d, ensuing, at $10\frac{1}{2}$ o'clock A.M. A society of the Alumni will then be formed.

The Twenty-Fifth Annual Session of the College will be introduced at $7\frac{1}{2}$ P.M., by an address from Moses Gunn, A.M., M.D., Professor of Surgery, and the exercises closed by ceremonies of a social and festive character.

The JOURNAL does not hesitate to say that the Alumni of the College, practitioners and students, will be welcomed to the most magnificent lecture-rooms on the continent, by warm hearts and cordial greetings. Old friendships will be revived and strengthened, and new ones formed. It is intended that the new college shall be a Mecca, to which, year by year, the Alumni of "Old Rush" shall gladly come up to assist in welding new links in the professional chain, infinitely stronger than the cold-blooded Trades-Unions, which some think the only bond of professional union. Come one—come all.

### *To Subscribers.*

Bills have been sent, as per published rates, to all subscribers who have not paid in advance for the JOURNAL. Mistakes will be cheerfully rectified, when pointed out, and full faith always given to the statements of our patrons. The books have been placed in the hands of a thoroughly competent bookkeeper, and order is rising from the chaos in which the present editor found affairs on remounting the tripod. Upon the conclusion of the *Endoscope*, space will be found in each number for a careful digest of current medical news, abstracts, items, etc. Lack of space has temporarily prevented the insertion of this chapter, which, hereafter, we hope to make one of large interest to our readers.

